# Improving the Quality, Accuracy and Consistency of Capacity Assessments Across Inpatient Psychiatric Wards at the Harbour (FC Locality)

**DOI:** 10.1192/bjo.2026.11394

**Published:** 2026-06-30

**Authors:** Claire-Marie Hosein

**Affiliations:** Lancashire and South Cumbria NHS Foundation Trust, Preston, United Kingdom

## Abstract

**Aims:**

To improve the quality, accuracy, and consistency of capacity assessments and their documentation in line with the Mental Capacity Act (2005) across inpatient psychiatric wards at The Harbour. The project aimed to ensure assessments were decision specific, clearly reasoned, and compliant with statutory standards, targeting at least 80% of assessments meeting all required components within 12 weeks following a targeted educational intervention.

**Methods:**

This quality improvement audit was conducted across all wards at The Harbour, a 154-bed mental health hospital, including general adult, older adult, dementia, and psychiatric intensive care wards. A proportional, representative sample of 40 capacity assessments completed by resident doctors was randomly selected from the Trust’s Clinical Information System using a computer-generated randomisation function. Baseline data were collected over two weeks, with the same process applied post-intervention.

Assessments were audited using a bespoke toolkit based on the Trust’s SoP: MCA Policy and Deprivation of Liberty Safeguards Procedure, the Mental Capacity Act (2005), MCA Code of Practice (2007), and NICE NG97 Dementia (2018), providing measurable standards for documentation, reasoning, and functional testing.

In week three, resident doctors received a 30-minute targeted educational session covering MCA principles, diagnostic and functional testing, decision and time specificity, and correct completion of the Trust’s Capacity Assessment template. Reinforcement occurred through multidisciplinary discussions, handovers, and optional quick-reference guidance. A re-auditof 40 assessments was conducted in weeks nine and ten, with analysis and reporting completed in weeks eleven and twelve.

**Results:**

Baseline audit revealed consistent documentation of decision and time specificity (100%), but lower compliance in key areas: diagnostic test (77.5%), functional components: understand (70%), retain (70%), use/weigh (70%), communicate (70%); clinical reasoning (60%), correct template use (67.5%), and decision-specific framing (60%). Capacity outcome was explicitly stated in 92.5% of assessments.

Post-intervention audit showed marked improvements: diagnostic test documentation 95%; functional components: understand 92.5%, retain 90%, use/weigh 90%, communicate 100%; clinical reasoning, template use, and decision-specific framing all rose to 92.5%; with capacity outcome 97.5%.

**Conclusion:**

A brief, targeted educational intervention substantially improved the quality, accuracy, and consistency of capacity assessments across inpatient psychiatric wards. Improvements in documentation, reasoning, template use, and decision-specific framing enhanced decision specificity and legal defensibility. By increasing familiarity with statutory requirements and structured templates, this low-cost, scalable intervention supports patient autonomy, strengthens clinician confidence, and can be readily applied in other psychiatric services where variability in capacity assessment persists.

